# A Stationary North-Finding Scheme for an Azimuth Rotational IMU Utilizing a Linear State Equality Constraint

**DOI:** 10.3390/s150204368

**Published:** 2015-02-13

**Authors:** Huapeng Yu, Hai Zhu, Dayuan Gao, Meng Yu, Wenqi Wu

**Affiliations:** 1 Department of Navigation and Communication, Navy Submarine Academy, Qingdao 266042, China; E-Mails: seapeter@163.com (H.Z.); dygao@263.net (D.G.); 2 College of Aerospace Science and Engineering, National University of Defense Technology, Changsha 410073, China; E-Mail: yumeng4517@gmail.com; 3 College of Mechatronics Engineering and Automation, National University of Defense Technology, Changsha 410073, China; E-Mail: wenqiwu_lit@nudt.edu.cn

**Keywords:** rotational IMU, north-finding, Kalman filter, stochastic observability

## Abstract

The Kalman filter (KF) has always been used to improve north-finding performance under practical conditions. By analyzing the characteristics of the azimuth rotational inertial measurement unit (ARIMU) on a stationary base, a linear state equality constraint for the conventional KF used in the fine north-finding filtering phase is derived. Then, a constrained KF using the state equality constraint is proposed and studied in depth. Estimation behaviors of the concerned navigation errors when implementing the conventional KF scheme and the constrained KF scheme during stationary north-finding are investigated analytically by the stochastic observability approach, which can provide explicit formulations of the navigation errors with influencing variables. Finally, multiple practical experimental tests at a fixed position are done on a postulate system to compare the stationary north-finding performance of the two filtering schemes. In conclusion, this study has successfully extended the utilization of the stochastic observability approach for analytic descriptions of estimation behaviors of the concerned navigation errors, and the constrained KF scheme has demonstrated its superiority over the conventional KF scheme for ARIMU stationary north-finding both theoretically and practically.

## 1. Introduction

The rotational inertial measurement unit (IMU) high precision inertial navigation system (INS) concept has been developed for use in long-term scenarios [1–4]. Due to its advantage in reducing the effect of gyroscope drift, there is growing interest in the navigation community for use of the rotational IMU concept with various gyroscopes applications [1–12]. Nowadays, many techniques are in use, with approaches such as single-axis rotation [1,3,4,6–11], dual-axis rotation [1,5,12], and triple-axis rotation [2] being the approaches of choice.

Generally, the single-axis rotation approach applies the azimuth rotational motion periodically to the IMU. Different schemes can be implemented for single-axis rotation, such as flip/dwell schemes [3,4], continuously rotating schemes [8,11,13], reciprocating rotation schemes [9,10], and so on. In the subsequent sections of this paper, an IMU with the continuously rotating scheme is called the azimuth rotational IMU (ARIMU). As it behaves with some specific characteristics, comprehensive research on the ARIMU is of great significance. From a mathematical point of view, Britting proved that continuous rotation offers an advantage in attenuating the effects of time-correlated random drift change and the operational environment is significantly better for the gyro during rotation [8]. An experimental method based on the fast orthogonal search for a practical observation model to separate angle random walk error from the RLG measured data on a turntable with continuous rotation was proposed in [11]. Theoretical explanations of the principle of restraining navigation errors by continuous rotation based on the navigation error equation were presented in [13].

In many practical applications, the azimuth determination requirements are high accuracy and short time [5–8,14–17]. The Kalman filter (KF) has always been used to provide self-calibration and to improve azimuth performance under practical conditions [2,3,6,8–10]. An observation model that includes north and east velocities and geographic frame east rate measurements was presented and illustrated by simulated IMU date in [16]. To improve self-alignment scheme for a strapdown INS in near stationary conditions, a nonlinear augmented measurement-based observation model was proposed in [18]. A rigorous analytical method of incorporating state equality constraints in the Kalman filter was developed to improve the prediction accuracy of the filter in [19]. These literatures are all very instructive to analysts concerned with azimuth determination problems.

Although a considerable amount of research on determining the azimuth angle has been done for the ARIMU [3–10,13], they did not pay much attention to the estimation behaviors (e.g., convergence rate) of navigation errors when implementing a Kalman filter. Taking advantage of its basic properties of intuitive linear-algebraic characterizations of the stochastic observability, azimuth behaviors during in-flight alignment when several characteristic maneuvers are performed were investigated and explicit findings were obtained analytically with simple models in [14,15].

The major thrusts of this paper are to provide a novel rapid north-finding filtering scheme for the ARIMU on a stationary base using a state equality constraint and to extend the utilization of the stochastic observability approach for analytic descriptions of estimation behaviors of the concerned navigation errors. For illustrative purposes, a postulate system, the principal idea of which is close to that of [9,10], was taken as the experimental set-up.

The remainder of the paper is organized as follows: in Section 2, an error dynamic model for the ARIMU and a conventional observation model for stationary north-finding are presented, followed by formulation of a linear state equality constraint and a proposed constrained KF for stationary north-finding. Section 3 describes the analytical solution for characterizing estimation behaviors of the concerned navigation errors during stationary north-finding based on the stochastic observability approach. Results and discussions of experimental verification are given in Section 4. Conclusions are drawn in Section 5.

## 2. Error Dynamic Model for the ARIMU and the State Equality Constraint

### 2.1. Modeling Error Dynamics for the ARIMU

In this subsection, a commonly used navigation error dynamic model is presented. For the reduction of computation load when applying stochastic observability analysis, the error dynamic equations in the *ψ* formulation will be introduced in this study.

**Figure 1.**
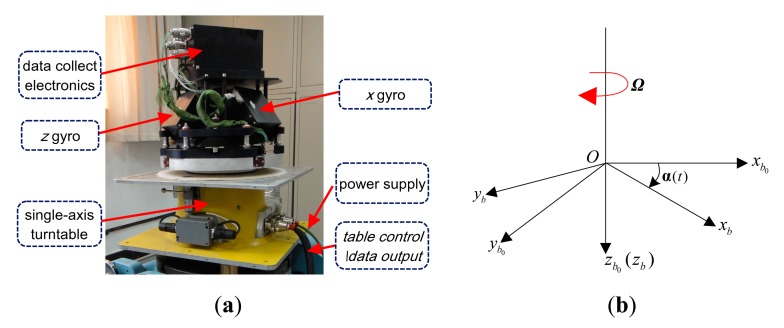
The experimental set-up and definition of coordinate frames in the ARIMU. (**a**) The experimental set-up; (**b**) Definition of coordinate frames in the ARIMU.

Figure 1 depicts the experimental set-up and a diagrammatic sketch of the coordinate frames in the ARIMU. The IMU contains a three-axis ring laser gyro (RLG) triad, a three-axis vibrating quartz accelerometer triad and their associated electronics. We denote by *b* the IMU fixed frame, and the *z_b_* axis lies along the turntable shaft center and downwards vertically to the turntable plane. As the IMU is rigidly fixed on a continuously rotational turntable, the *b* frame is rotated with the turntable rotation [11].

For the sake of convenience, we define the *b*_0_ frame when the *x_b_* axis coincides with the turntable null indicator. Then, we have:

(1) $$C_{b}^{b_{0}}=\left[\begin{array}{ccc} \cos\alpha \left(t\right) & -\sin\alpha \left(t\right) & 0\\ \sin\alpha \left(t\right) & \cos\alpha \left(t\right) & 0\\ 0 & 0 & 1 \end{array}\right]$$

where ***α*** represents the rotation angle of the *x_b_* axis with respect to the turntable null indicator, and $\alpha \left(t\right)=\int_{0}^{t}\Omega dt$. ***Ω*** denotes the turntable rotation rate.

As the *b*_0_ frame is turntable fixed, $C_{b_{0}}^{n}$remains constant on stationary base. In practice, the *b*_0_ frame is approximately leveled, the roll and pitch angles are small enough, it yields:

(2) $$C_{b_{0}}^{n}=\left[\begin{array}{ccc} \cos\varphi & -\sin\varphi & \theta \sin\varphi +\gamma \cos\varphi \\ \sin\varphi & \cos\varphi & -\theta \cos\varphi +\gamma \sin\varphi \\ -\gamma & \theta & 1 \end{array}\right]$$

where *θ*, *γ*, *φ* denote the roll, pitch and azimuth angles, respectively. The objective of north-finding is to determine *φ* in this study.

Thus, a transformation from the *b* frame to the *n* frame may be expressed as the product of Equations (1) and (2), we may write:

(3) $$C_{b}^{n}=C_{b_{0}}^{n}C_{b}^{b_{0}}=\left[\begin{array}{ccc} C_{11} & C_{12} & C_{13}\\ C_{21} & C_{22} & C_{23}\\ C_{31} & C_{32} & C_{33} \end{array}\right]$$

Neglecting other error sources such as factor error, temperature effect, and so on, gyro triad biases and accelerometer triad biases are assumed to be constant. Then, basic navigation error equations in ***ψ*** form expressed in the north-east-down (NED) coordinate system are as follows [16,20,21]:

(4) $$\dot{\psi }=\psi \times \left(\omega_{en}^{n}+\omega_{ie}^{n}\right)-C_{b}^{n}\varepsilon^{b}$$

(5) $$\delta \dot{v}=f^{n}\times \psi -\left(\omega_{en}^{n}+2\omega_{ie}^{n}\right)\times \delta v+C_{b}^{n}\nabla^{b}$$

(6) $${\dot{\varepsilon }}^{b}=0$$

(7) $${\dot{\nabla }}^{b}=0$$

where *i*, *e*, *n*, represent the inertial frame, the Earth frame, and the local geographic navigation frame, respectively. ***ψ*** represents the attitude error vector, and ***ψ*** = [*ψ_N_ψ_E_ψ_D_*]^T^. *δ****v*** represents the velocity error vector expressed in the *n* frame. $\omega_{ie}^{n}$is the turn rate of the Earth frame with respect to the *i* frame expressed in the *n* frame, and $\omega_{en}^{n}$is the turn rate of the *n* frame with respect to the *e* frame. ***f*** represents a measure of the specific force acceleration. ***ε*** and ∇ are defined as gyro triad bias vector and accelerometer triad bias vector, respectively.

The error items including attitude error components of ***ψ***, north velocity error *δ**v**_N_*, east velocity error *δ**v**_E_*, gyro bias components of ***ε****^b^*, accelerometer bias acting about the *x_b_* axis $\nabla_{x}^{b}$, and accelerometer bias acting about the *y_b_* axis $\nabla_{y}^{b}$are taken into account in the state update equations of the implemented Kalman filters. Meanwhile, $\omega_{en}^{n}$is equal to **0** when the *b*_0_ frame is at rest. Hence, error dynamic model for the ARIMU is given by:

(8) $$\begin{array}{cc} \dot{x}\left(t\right) & =\left[\begin{array}{cc} F & G\left(t\right)\\ 0_{5\times 5} & 0_{5\times 5} \end{array}\right]\;x\left(t\right)+\left[\begin{array}{c} w_{f}\left(t\right)\\ 0_{5\times 1} \end{array}\right]\\ & \begin{array}{cc} =Ax\left(t\right)+w\left(t\right), & w\left(t\right)\sim N\left(0,Q\right) \end{array} \end{array}$$

where $x={\left[\begin{array}{cc} \begin{array}{ccccc} \psi_{N} & \psi_{E} & \psi_{D} & \delta v_{N} & \delta v_{E} \end{array} & \begin{array}{ccccc} \varepsilon_{x}^{b} & \varepsilon_{y}^{b} & \varepsilon_{z}^{b} & \nabla_{x}^{b} & \nabla_{y}^{b} \end{array} \end{array}\right]}^{T}$, $w_{f}={\left[\begin{array}{ccccc} w_{\psi N} & w_{\psi E} & w_{\psi D} & w_{\delta vN} & w_{\delta vE} \end{array}\right]}^{T}$.

Components of ***w****_f_* add small random walk quantities to *δ****v*** and ***ψ***. The matrix ***F*** takes the following form:

(9) $$F=\left[\begin{array}{cc} \begin{array}{ccc} 0 & \omega_{\text{ieD}}^{n} & 0\\ -\omega_{\text{ieD}}^{n} & 0 & \omega_{\text{ieN}}^{n}\\ 0 & -\omega_{\text{ieN}}^{n} & 0 \end{array} & \begin{array}{cc} 0 & 0\\ 0 & 0\\ 0 & 0 \end{array}\\ \begin{array}{ccc} 0 & g & 0\\ -g & 0 & 0 \end{array} & \begin{array}{cc} 0 & 2\omega_{\text{ieD}}^{n}\\ -2\omega_{\text{ieD}}^{n} & 0 \end{array} \end{array}\right]$$

Similarly, we have:

(10) $$G(t)=\left[\begin{array}{cc} -C_{b}^{n} & \begin{array}{cc} 0_{3✕1} & 0_{3✕1} \end{array}\\ \begin{array}{c} 0_{1✕3}\\ 0_{1✕3} \end{array} & \begin{array}{cc} C_{11} & C_{12}\\ C_{21} & C_{22} \end{array} \end{array}\right]$$

For stationary north-finding, the position has been initialized and the ARIMU uses zero velocity updates, *v_N_* and *v_E_* are supposed to be zero [20,22]. Therefore, *δv_N_* and *δv_E_* are chosen as the measured quantities conventionally. The conventional observation model is given by:

(11) $$\begin{array}{ll} z\left(t\right) & =\left[\begin{array}{cc} \begin{array}{cc} 0_{2\times 3} & I_{2\times 2} \end{array} & \begin{array}{c} 0_{2\times 5} \end{array} \end{array}\right]\;x\left(t\right)+\left[\begin{array}{c} v_{1}\left(t\right)\\ v_{2}\left(t\right) \end{array}\right]\\ & \begin{array}{cc} =Hx\left(t\right)+v\left(t\right), & v\left(t\right)\sim N\left(0,R\right) \end{array} \end{array}$$

where ***I***_2×2_ is a second order identity matrix. ***v***(*t*) denotes the measurement noise vector. Main noise sources of the measurement noise are vibratory motions and small position displacements due to human activity, such as disturbance, loading, fuelling, and boarding. Sometimes, the measurement noise covariance is artificially increased to prevent instability of the Kalman filter [16,22]. In addition, it is assumed that ***w***(*t*) and ***v***(*t*) are uncorrelated [20,22].

Then, optimal estimation of the navigation error states can be achieved based on the augmented error dynamic model (8) and the conventional observation model (11) using a Kalman filter, which we called the conventional KF scheme.

### 2.2. The State Equality Constraint Formulation and the Proposed constrained KF Equations

By analyzing the characteristics of the ARIMU on a stationary base, we obtain a linear state equality constraint to modify the conventional observation model in this subsection. Theoretically, angular rate measured by the gyro triad under stationary conditions can be expressed in the *b* frame, that is:

(12) $$\omega_{ib}^{b}=\omega_{nb}^{b}+C_{n}^{b}\omega_{ie}^{n}$$

However, as mentioned before, gyroscopes are unfortunately subject to errors such as constant bias, bias stability, temperature effect, and so on. Considering only the gyro constant bias exists as in Equation (6) in the true environment, angular rate measured by the gyro triad under stationary condition, denoted by ${\tilde{\omega }}_{ib}^{b}$, is given by:

(13) $${\tilde{\omega }}_{ib}^{b}=\omega_{nb}^{b}+C_{n}^{b}\omega_{ie}^{n}+\varepsilon^{b}$$

During stationary north-finding, the estimated direction cosine matrix ${\tilde{C}}_{b}^{n}$ is used for attitude update computation. Therefore, computed angular rate in the realized computer program can be written as:

(14) $${\hat{\omega }}_{ib}^{b}=\omega_{nb}^{b}+{\tilde{C}}_{n}^{b}\omega_{ie}^{n}+\varepsilon^{b}$$

where ${\tilde{C}}_{b}^{n}=\left(I-\psi \times \right)C_{b}^{n}$.

Combining Equations (12), (13), and (14), we obtain:

(15) $$\begin{array}{c} \delta \omega_{ib}^{b}={\tilde{\omega }}_{ib}^{b}-\left(\omega_{nb}^{b}+{\tilde{C}}_{n}^{b}\omega_{ie}^{n}\right)=\left(C_{n}^{b}-{\tilde{C}}_{n}^{b}\right)\omega_{ie}^{n}+\varepsilon^{b}\\ =-C_{n}^{b}\left[\psi \times \right]\omega_{ie}^{n}+\varepsilon^{b} \end{array}$$

Ignoring small quantity product terms, we then have a state equality constraint equation:

(16) $$\delta \omega_{\text{ibz}}^{b}=\omega_{\text{ieN}}^{n}\psi_{E}+\varepsilon_{z}^{b}$$

As can be seen in Equation (16), both of *ψ_E_* and $\varepsilon_{z}^{b}$ are navigation error states of the error dynamic model (8). Therefore, we can incorporate the linear state equality constraint (16) in the conventional observation model (11). Then, the modified observation model may be expressed as:

(17) $$\begin{array}{ll} \bar{z}\left(t\right) & =\left[\begin{array}{c} z\left(t\right)\\ \delta \omega_{\text{ibz}}^{b} \end{array}\right]=\left[\begin{array}{cc} \begin{array}{cccc} 0_{2✕1} & 0_{2✕1} & 0_{2✕1} & I_{2✕2} \end{array} & \begin{array}{ccc} 0_{2✕2} & 0_{2✕1} & 0_{2✕2} \end{array}\\ \begin{array}{cccc} 0 & \omega_{\text{ieN}}^{n} & 0 & 0_{1✕2} \end{array} & \begin{array}{ccc} 0_{1✕2} & 1 & 0_{1✕2} \end{array} \end{array}\right]\;x\left(t\right)+\left[\begin{array}{c} \begin{array}{c} v\left(t\right) \end{array}\\ \begin{array}{c} w_{zb} \end{array} \end{array}\right]\\ & \begin{array}{ll} =\bar{H}x\left(t\right)+\bar{v}\left(t\right), & \bar{v}\left(t\right)\sim N\left(0,\bar{R}\right) \end{array} \end{array}$$

where *w_zb_* represents the computed angular rate measurement noise acting about the *z_b_* axis. Using gyro triad measured data and turntable rotation information, the added linear measured quantity $\delta \omega_{\text{ibz}}^{b}$can be computed to fuse the effects of the two error states into one. Meanwhile, it should be noted that, although they are neglected above in the basic navigation error equations for simplification, error sources whose influencing effects are equivalent to the gyro bias $\varepsilon_{z}^{b}$have been taken into account by the added measured quantity $\delta \omega_{\text{ibz}}^{b}$ and will be estimated in the filtering process. Taking gyro triad factor errors for example, their influencing effects in the gyro triad measured data equals the gyro triad biases when the turntable rotation rate is constant and the ARIMU is stationary, so the estimated value $\varepsilon_{z}^{b}$ will eventually include their equivalent component on the *z_b_* axis gyro [22].

Consequently, we propose a constrained KF using the augmented error dynamic model (8) and the modified observation model (17), which is called the constrained KF scheme in the following sections.

## 3. Stochastic Observability Measures

The stochastic observability approach, which connects the observability analysis with investigation of convergence rates of the concerned navigation errors when implementing a Kalman filter, has demonstrated its effectiveness in successful applications to solving in-flight alignment problems in [14,15]. With the stochastic observability approach, we will provide in this section an analytical solution for characterizing estimation behaviors of the navigation errors during stationary north-finding. Meanwhile, estimation performances of the concerned navigation errors utilizing the conventional KF scheme and the constrained KF scheme are compared. The following analytic and numerical computations are carried out with Wolfram Mathematica 7.0 tool.

### 3.1. Stochastic Observability

Before entering stochastic observability analysis, some necessary explanations and definitions of observability of linear systems adopted in this study are introduced. For purpose of extending known definitions to stationary north-finding problem, we will adopt the following meanings.

In the absence of process noise and *a priori* information, the solution to the *continuous* system matrix Riccati equation in ***P***^−1^ is given by [14,23]:

(18) $$P^{-1}\left(t\right)=\int_{0}^{t}\Phi^{T}\left(\tau ,t\right)H^{T}\left(\tau \right)R^{-1}\left(\tau \right)H\left(\tau \right)\Phi \left(\tau ,t\right)d\tau$$

where **Φ**(*t*, *τ*) represents the transition matrix corresponding to ***A***, and the integral on the right-side of Equation (18) is the *stochastic observability matrix* of the linear system. Based on characteristics of the state transition matrix [23], we have:

(19) $$\Phi \left(t,\tau \right)=\left[\begin{array}{cc} \begin{array}{c} e^{F\left(t-\tau \right)} \end{array} & \begin{array}{c} \int_{\tau }^{t}e^{F\left(t-\lambda \right)}G\left(\lambda \right)d\lambda \end{array}\\ \begin{array}{c} 0_{5\times 5} \end{array} & \begin{array}{c} I_{5\times 5} \end{array} \end{array}\right]$$

Along with the use of the stochastic observability approach, special attention should be paid to the following points:

(a) According to the precondition of Equation (18), it should be noted that stochastic observability analyses in the following subsections are performed without considerations of process noise and *a priori* knowledge of the initial states.
(b) The system is *uniformly completely observable* when the *stochastic observability matrix* is positive definite and bounded for some *t* > 0 [23].

As indicated in Equation (18), information (decrease the estimation error variance) about states that are initially completely unknown may be acquired through processing measurements. Besides, with error covariance matrix computations, convergence rates can be analytically investigated to maintain good physical insight into the estimation behaviors of the navigation errors [14,23].

### 3.2. Analytic Descriptions of Estimation Behaviors of Navigation Errors during North-Finding

To characterize estimation behaviors of the concerned navigation errors clearly, acquiring analytic relationships between the navigation errors and correlated influencing variables are the most essential solutions, as is one of our main purposes in this study. In this subsection, we focus on the azimuth error and the *z_b_* axis gyro error, which are the two most crucial state variables in the error dynamic model (8). The diagonal elements of the covariance matrix ***P***(*t*) indicate estimates of the magnitudes of the navigation errors. Thus, the third and eighth diagonal elements of the covariance matrix ***P***(*t*), which are denoted as *P*_(3,3)_(*t*) and *P*_(8,8)_(*t*), may be regarded as the quantitative measures of observability of the azimuth error and the *z_b_* axis gyro error [8,14–16,20,21].

Suppose that total time for stationary north-finding is *t_s_*, we have ***P***(*t_s_*) for the conventional KF scheme from Equation (18):

(20) $$P^{0}\left(t_{s}\right)={\left[\int_{0}^{t_{s}}\Phi^{T}\left(\tau ,t_{s}\right)H^{T}{\left[\begin{array}{cc} r_{0}^{2} & 0\\ 0 & r_{0}^{2} \end{array}\right]}^{-1}H\Phi \left(\tau ,t_{s}\right)d\tau \right]}^{-1}$$

where variances of *v*_1_(*t*) and *v*_2_(*t*) are assumed to be equal to the same value, and the value of diagonal elements of ***R*** are denote by *r*_0_^2^.

Similarly, ***P***(*t_s_*) for the constrained KF scheme is given by:

(21) $$P^{1}\left(t_{s}\right)={\left[\int_{0}^{t_{s}}\Phi^{T}\left(\tau ,t_{s}\right){\bar{H}}^{T}{\left[\begin{array}{ccc} r_{0}^{2} & 0 & 0\\ 0 & r_{0}^{2} & 0\\ 0 & 0 & r_{g}^{2} \end{array}\right]}^{-1}\bar{H}\Phi \left(\tau ,t_{s}\right)d\tau \right]}^{-1}$$

where variance of *w_zb_*, which is equal to the value of the third diagonal element of ***R̄***, is denoted by *r*_0_^2^.

As numerical values of the diagonal elements of the error covariance matrix ***P*** at time *t_s_* implies ultimate estimation accuracies of the navigation errors, we can investigate improvement of the constrained KF scheme with respect to the conventional KF scheme by computing ***P***(*t_s_*). Due to the complex products of trigonometric function in $C_{b}^{n}$ computation, complete symbolic computation on ***P***(*t_s_*) still require computer with rather high performance. Therefore, it is difficult to derive general conditions for the analytic solution. To circumvent this problem of acquiring analytic descriptions of estimation behaviors of the navigation errors during stationary north-finding, we compute ***P***(*t_s_*) with certain influencing variable analytically to reduce computation load.

In the simplified ***P***(*t_s_*) calculation, the WGS-84 model is applied, and the total time *t_s_* is 600 s. The *b*_0_ frame is supposed to be coincidence with the *n* frame. Besides, it is assumed that the stationary north-finding be carried out at a latitude at sea level of 0.492535 rad. Then, there remains only the measurement noise standard deviations *r*_0_ and *r_g_* and the turntable rotation rate ***Ω*** as the influencing variables.

#### 3.2.1. The Effects of the Measurement Noises on Final Azimuth Error and Final *z_b_* Axis Gyro Error

To investigate the effects of the measurement noises on final azimuth error and final *z_b_* axis gyro error at 600 s analytically, the turntable rotation rate must be determined. For consistency with the subsequent experimental verification, ***Ω*** is set to be 0.6981 rad/s (or 40 deg/s).

Combining Equations (11), (19) and (20), one can obtain the numerical value of ***P***^0^ (*t_s_*). Then, analytic description of final azimuth error with *r*_0_ for the conventional KF scheme worked out to be:

(22) $$\sqrt{P_{\left(3,3\right)}^{0}\left(t_{s}\right)}=0.014484267327r_{0}$$

And, analytic description of final *z_b_* axis gyro error with *r*_0_ for the conventional KF scheme is given by:

(23) $$\sqrt{P_{\left(8,8\right)}^{0}\left(t_{s}\right)}=3.173625686813112\times 10^{-5}r_{0}$$

Similarly, one can obtain the numerical value of ***P***^1^ (*t_s_*) by combining Equations (17), (19) and (21). Then, the analytical description of the variance of final azimuth error with *r*_0_ and *r_g_* for the constrained KF scheme may be expressed as follows:

(24) $$\begin{array}{l} P_{\left(3,3\right)}^{1}\left(t_{s}\right)=\frac{\sum_{i=1}^{6}a_{i}r_{0}^{2\left(7-i\right)}r_{g}^{2i}}{\sum_{j=0}^{6}b_{j}r_{0}^{2\left(6-j\right)}r_{g}^{2j}}\\ =\frac{3.58913\times 10^{-21}r_{0}^{12}r_{g}^{2}+329.673r_{0}^{10}r_{g}^{4}+6.42751\times 10^{21}r_{0}^{8}r_{g}^{6}+7.7487\times 10^{29}r_{0}^{6}r_{g}^{8}-4.18141\times 10^{36}r_{0}^{4}r_{g}^{10}-2.74098\times 10^{46}r_{0}^{2}r_{g}^{12}}{1.51838\times 10^{-37}r_{0}^{12}+2.15786\times 10^{-11}r_{0}^{10}r_{g}^{2}+1.9767\times 10^{12}r_{0}^{8}r_{g}^{4}+1.10083\times 10^{27}r_{0}^{6}r_{g}^{6}+6.92068\times 10^{34}r_{0}^{4}r_{g}^{8}+4.84689\times 10^{44}r_{0}^{2}r_{g}^{10}+1.96838\times 10^{52}r_{0}^{12}} \end{array}$$

The analytical description of the variance of final *z_b_* axis gyro error with *r*_0_ and *r_g_* for the constrained KF scheme can be obtained as:

(25) $$\begin{array}{l} P_{\left(8,8\right)}^{1}\left(t_{s}\right)=\frac{\sum_{i=1}^{6}h_{i}r_{0}^{2\left(7-i\right)}r_{g}^{2i}}{\sum_{j=0}^{6}l_{j}r_{0}^{2\left(6-j\right)}r_{g}^{2j}}\\ =\frac{1.04024\times 10^{-32}r_{0}^{12}r_{g}^{2}+4.71324\times 10^{-6}r_{0}^{10}r_{g}^{4}+1.57526\times 10^{10}r_{0}^{8}r_{g}^{6}+1.83084\times 10^{24}r_{0}^{6}r_{g}^{8}-1.12079\times 10^{32}r_{0}^{4}r_{g}^{10}-8.05148\times 10^{41}r_{0}^{2}r_{g}^{12}}{1.51838\times 10^{-37}r_{0}^{12}+2.15786\times 10^{-11}r_{0}^{10}r_{g}^{2}+1.9767\times 10^{12}r_{0}^{8}r_{g}^{4}+1.10083\times 10^{27}r_{0}^{6}r_{g}^{6}+6.92068\times 10^{34}r_{0}^{4}r_{g}^{8}+4.84689\times 10^{44}r_{0}^{2}r_{g}^{10}+1.96838\times 10^{52}r_{0}^{12}} \end{array}$$

To compare the estimation behaviors of the concerned navigation errors by the above two filtering schemes, covariance calculations can be accomplished numerically based on Equations (22)–(25) to obtain the necessary information.

**Figure 2.**
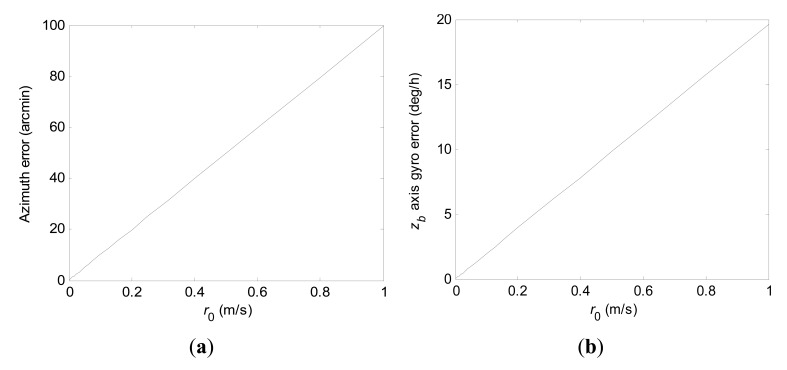
1-σ values of final azimuth error and final *z_b_* axis gyro error at 600 s computed according to *r*_0_ when using the conventional KF scheme. (**a**) Azimuth error; (**b**) *z_b_* axis gyro erro.

Figure 2 depicts 1-σ values of the final azimuth error and final *z_b_* axis gyro error at 600 s computed according to *r*_0_ when using the conventional KF scheme. The numerical range of *r*_0_ is 0.001 m/s to 1 m/s. From Figure 2, it is clearly seen that 1-σ values of final azimuth error and final *z_b_* axis gyro error are theoretically directly proportional to *r*_0_ when ***Ω*** is set.

**Figure 3.**
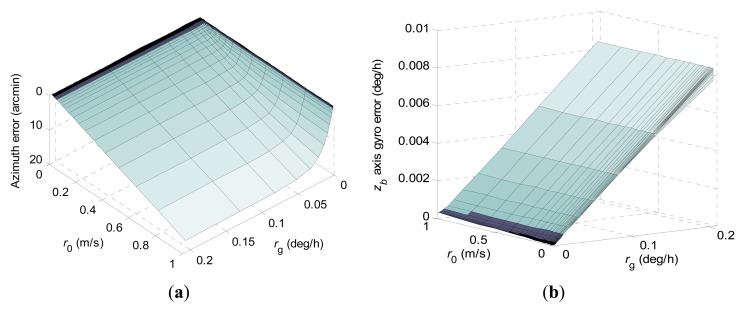
1-σ values of final azimuth error and final *z_b_* axis gyro error at 600 s computed according to *r*_0_ and *r_g_* when using the constrained KF scheme. (**a**) Azimuth error; (**b**) *z_b_* axis gyro erro.

1-σ values of the final azimuth error and final *z_b_* axis gyro error at 600 s computed according to *r*_0_ and *r_g_* when using the constrained KF scheme are shown in Figure 3. The numerical range of *r*_0_ is the same as in the conventional KF scheme, and the numerical range of *r_g_* is 2 × 10^−5^ deg/h to 0.2 deg/h.

In Figure 3 it can be seen that the state equality constraint adopted in the constrained KF scheme has a great influence on the estimation behaviors of the concerned navigation errors. Obviously, the constrained KF scheme has achieved much better north-finding performance than the conventional KF scheme. The 1-σ value of the final azimuth error is theoretically proportional to *r*_0_ when ***Ω*** and *r_g_* are set. However, the 1-σ value of the final azimuth error behaves with a nonlinear variation with *r_g_* when ***Ω*** and *r*_0_ are set.

#### 3.2.2. The Effects of the Turntable Rotation Rate on Final Azimuth Error and Final *z_b_* Axis Gyro Error

The measurement noise standard deviation *r*_0_ will be determined likewise in this subsection for practical implementation. From an empirical standpoint, *r*_0_ is set to be 0.01 m/s on a stationary base.

**Figure 4.**
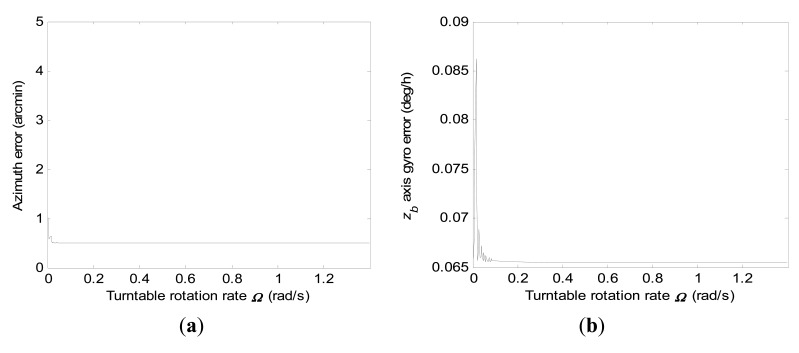
1-σ values of final azimuth error and final *z_b_* axis gyro error at 600 s computed according to turntable rotation rates ranging from 0.0003 rad/s to 1.4 rad/s when using the conventional KF scheme. (**a**) Azimuth error; (**b**) *z_b_* axis gyro erro.

Analytical descriptions of the final azimuth error and final *z_b_* axis gyro error at 600 s with ***Ω*** for the conventional KF scheme take a rather complex form including the items ***Ω***, trigonometric functions with 600 Ω, and so on. Therefore, we can not obtain simple expressions for $P_{\left(3,3\right)}^{0}\left(t_{s}\right)$ and $P_{\left(8,8\right)}^{0}\left(t_{s}\right)$ when *r*_0_ is set. Consequently, we only give the diagrammatic representations of 1-σ values of final azimuth error and final *z_b_* axis gyro error at 600 s by error covariance computations according to different turntable rotation rates for the conventional KF scheme, respectively, in Figure 4.

In Figure 4, it is illustrated that the final azimuth error and final *z_b_* axis gyro at 600 s error approach a steady value when the turntable rotation rate is high enough, which indicates that increasing the turntable rotation rate has little influence on improvement of the estimation accuracy of the concerned navigation errors at this moment.

**Figure 5.**
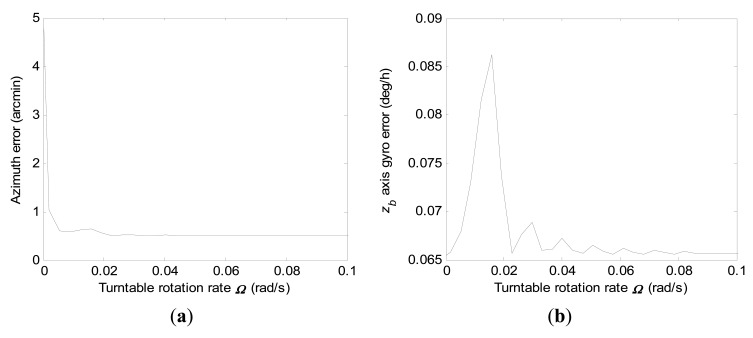
1-σ values of final azimuth error and final *z_b_* axis gyro error at 600 s computed according to turntable rotation rates ranging from 0.0003 rad/s to 0.1 rad/s when using the conventional KF scheme. (**a**) Azimuth error; (**b**) *z_b_* axis gyro erro.

The turntable rotation rate threshold is about 0.1 rad/s. 1-σ values of final azimuth error and final *z_b_* axis gyro error at 600 s computed according to turntable rotation rates ranging from 0.0003 rad/s to 0.1 rad/s for the conventional KF scheme are shown in Figure 5, respectively.

Figure 5 has given a more explicit indication, which yields that the final *z_b_* axis gyro error behaves with an oscillatory convergence with turntable rotation rate increases. However, it can be seen that final azimuth error approaches a steady value with little fluctuation when the turntable rotation rate increases.

**Figure 6.**
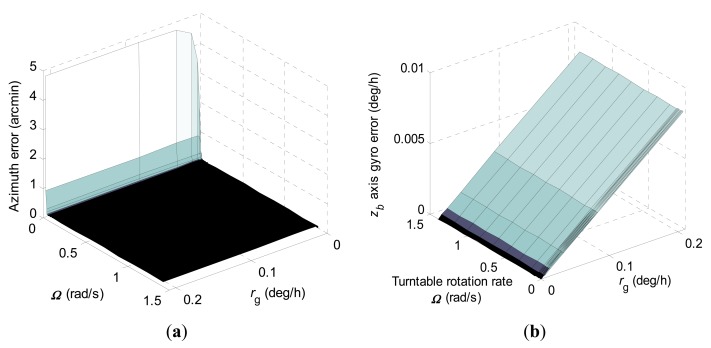
1-σ values of final azimuth error and final *z_b_* axis gyro error at 600 s computed according to different turntable rotation rates and *r_g_* when using the constrained KF scheme. (**a**) Azimuth error; (**b**) *z_b_* axis gyro erro.

Similarly, diagrammatic representations of the 1-σ values of the final azimuth error and final *z_b_* axis gyro error at 600 s by error covariance computations according to different turntable rotation rates and *r_g_* for the constrained KF scheme are shown in Figure 6, respectively.

In Figure 6, it can be seen that the final azimuth error and final *z_b_* axis gyro error have been decreased greatly even when *r_g_* approaches 0.2 deg/h. Meanwhile, the final azimuth error and final *z_b_* axis gyro error approach a steady value when the turntable rotation rate is high enough, and also the turntable rotation rate threshold is the same as in the conventional KF scheme.

**Figure 7.**
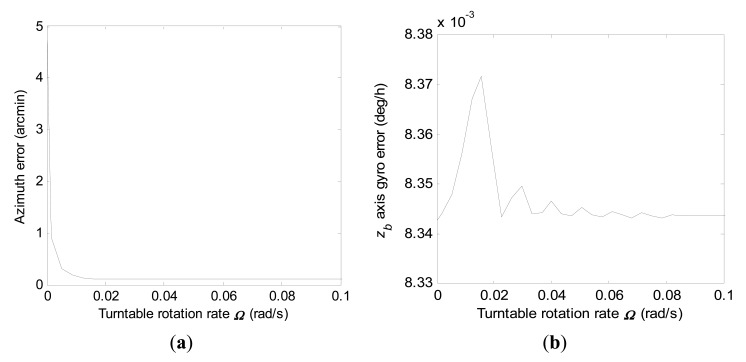
1-σ values of final azimuth error and final *z_b_* axis gyro error at 600 s computed according to turntable rotation rates ranging from 0.0003 rad/s to 0.1 rad/s when using the constrained KF scheme and *r_g_* is 0.2 deg/h. (**a**) Azimuth error; (**b**) *z_b_* axis gyro erro.

1-σ values of the final azimuth error and final *z_b_* axis gyro error at 600 s computed according to turntable rotation rates ranging from 0.0003 rad/s to 0.1 rad/s for the constrained KF scheme when *r_g_* is 0.2 deg/h are shown in Figure 7, respectively.

Compared to Figure 5, it can be seen in Figure 7 that the constrained KF scheme has improved the estimation performance of the azimuth error greatly. However, it should be noted that oscillatory convergence characteristics of final *z_b_* axis gyro error with turntable rotation rate increase still exists. Therefore, analysts dealing with ARIMU at low turntable rotation rates should pay attention to this phenomenon and need to split the difference between final azimuth error and final *z_b_* axis gyro error, which is similar to the idea expressed in [24].

#### 3.2.3. Convergence Rates of the Azimuth Error and the *z_b_* Axis Gyro Error

**Figure 8.**
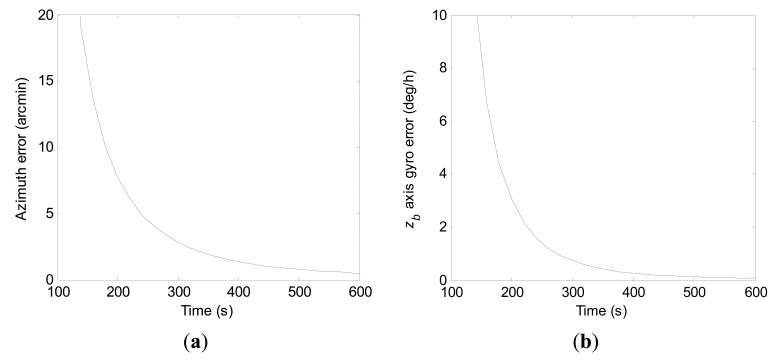
Convergence curves of the azimuth error and the *z_b_* axis gyro error within 600 s by the conventional KF scheme. (**a**) Azimuth error; (**b**) *z_b_* axis gyro erro.

By setting *r*_0_ and ***Ω*** to be 0.01 m/s and 0.6981 rad/s respectively, the convergence rates of the azimuth error and the *z_b_* axis gyro error during stationary north-finding by the two filtering schemes within total time *t_s_* are investigated in this subsection. Figure 8 gives convergence curves of the azimuth error and the *z_b_* axis gyro error with error covariance computations by Equation (20) within 600 s for the conventional KF scheme. From the numerical computation results, the magnitudes of the final azimuth error and the final *z_b_* axis gyro error at 600 s are 29.90 arc-seconds and 0.066 deg/h, respectively.

**Figure 9.**
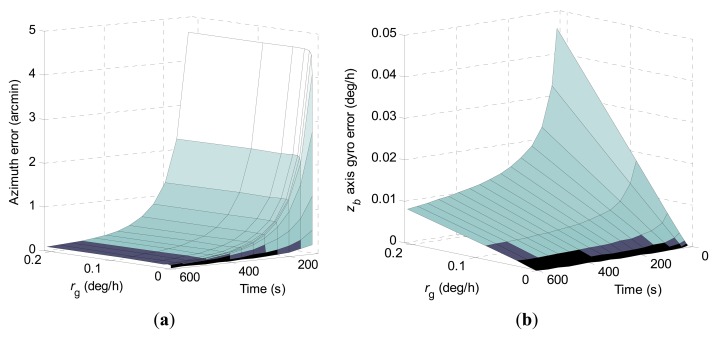
Convergence curves of the azimuth error and the *z_b_* axis gyro error within 600 s by the constrained KF scheme. (**a**) Azimuth error; (**b**) *z_b_* axis gyro erro.

Convergence curves of the azimuth error and the *z_b_* axis gyro error with error covariance computations by Equation (21) within 600 s for the constrained KF scheme are shown in Figure 9, respectively. It should be noted that the final *z_b_* axis gyro error at 600 s is directly proportional to *r_g_*. Therefore, the measurement noise *w_zb_* should be restrained by appropriate techniques to improve the north-finding performance. From Figure 9, it can be seen that the azimuth error and the *z_b_* axis gyro error converge with time faster when *r_g_* approaches the smaller value.

Compared to Figure 8, it is seen that convergence rates of the azimuth error and the *z_b_* axis gyro error by the constrained KF scheme are much faster than by the conventional KF scheme, even when *r_g_* is 0.2 deg/h. Meanwhile, from the error covariance computation results, the final azimuth error at 600 s can theoretically be reduced to just a few arc-seconds by the constrained KF scheme.

As illustrated above, extended application of the stochastic observability approach proposed in [14,15] for analytical description of a higher order error model has been accomplished successfully in this study. Ultimately, the above analytical depictions and graphic presentations of the stochastic observability analysis have provided important information showing that the constrained KF scheme displays a great estimation performance improvement over the conventional KF scheme.

## 4. Experimental Results of the Postulate System

The first performance evaluation of the rate-biased RLG in the postulate system, which utilizes the concept of the ARIMU, was given in [11]. The system has been developed to meet rapid north-finding challenges. Steps actually required to accomplish a rotation scheme of the postulate system are listed below:

(a) It can be seen in Equation (17) that, the measurement noise *w_zb_* existing in the added measured quantity $\delta \omega_{\text{ibz}}^{b}$ is composed of gyro triad measurement random errors and turntable rotation random errors. Therefore, a 2 milliseconds sample time of the IMU measured data and turntable rotation information and an angular resolution of 0.18″ of the turntable photoelectric angle encoder are implemented on the experimental platform, as can be confirmed to restrain the measurement noise *w_zb_* to low enough [11].
(b) According to the estimation results of the average angle random walk of the constant rate-biased RLG in the postulate system presented in [11], the turntable rotation rate should be larger than 40 deg/s to obviate the effect of lock-in at the low rotation rates. Besides, it helps little to improve stationary azimuth performance when the turntable rotation rate is higher than 0.1 rad/s. Thus, the turntable is determined to be continuously rotating at a rate of 40 deg/s.

### 4.1. Theoretical Analysis with Practical Considerations

As indicated earlier, stochastic observability analysis of the above two filtering schemes in Section 3 were carried out without consideration of process noise and *a priori* knowledge of the initial states. In practical applications, there is always a coarse azimuth determination phase before the fine north-finding filtering phase to provide the *a priori* knowledge of the initial states to match the applicability conditions of Equations (4) and (5). After the coarse azimuth determination phase, the 1-σ value of the azimuth error worked out to be 1.64°; 1-σ values of the roll and pitch angle errors are equal to the same value, which is 0.1°, assuming that both of the 1-σ values of the north and east velocity errors are 1 m/s. In the laboratory environment, the measurement noise standard deviation *r*_0_ is 0.01 m/s, and *r_g_* is 0.002 deg/h. The *a priori* knowledge of the initial states is given by

(26) $$P\left(0\right)=\text{diag}\left\{\begin{array}{cccccccccc} {\left(0.1{}^{\circ}\right)}^{2} & {\left(0.1{}^{\circ}\right)}^{2} & {\left(1.64{}^{\circ}\right)}^{2} & 1 & 1 & {\left(0.01{}^{\circ}/\text{h}\right)}^{2} & {\left(0.01{}^{\circ}/\text{h}\right)}^{2} & {\left(0.1{}^{\circ}/\text{h}\right)}^{2} & {\left(100\mu \text{g}\right)}^{2} & {\left(100\mu \text{g}\right)}^{2} \end{array}\right\}$$

With consideration of the *a priori* knowledge of the initial states, the solution to the *continuous* system matrix Riccati equation in *P*^−1^ can be written as [15]:

(27) $$P^{-1}\left(t\right)=\Phi^{T}\left(0,t\right)P^{-1}\left(0\right)\Phi \left(0,t\right)+\int_{0}^{t}\Phi^{T}\left(\tau ,t\right)H^{T}\left(\tau \right)R^{-1}\left(\tau \right)H\left(\tau \right)\Phi \left(\tau ,t\right)d\tau$$

**Figure 10.**
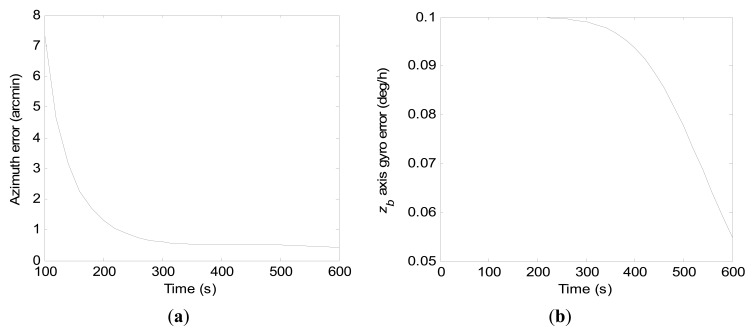
Convergence curves of the azimuth error and the *z_b_* axis gyro error within 600 s by the conventional KF scheme in practical application. (**a**) Azimuth error; (**b**) *z_b_* axis gyro erro.

**Figure 11.**
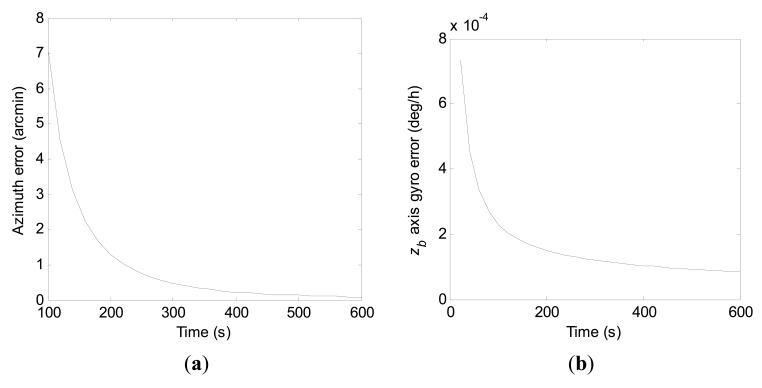
Convergence curves of the azimuth error and the *z_b_* axis gyro error within 600 s by the constrained KF scheme in practical application. (**a**) Azimuth error; (**b**) *z_b_* axis gyro erro.

Therefore, convergence rates of the azimuth error and the *z_b_* axis gyro error in the true environment can be obtained by error covariance computations with Equation (27) in a similar manner as in Section 3. With the *a priori* knowledge of the initial states, convergence curves of the azimuth error and the *z_b_* axis gyro error within 600 s for the conventional KF scheme and the constrained KF scheme in the practical application are shown in Figures 10 and 11, respectively.

Compared to Figure 8, it is obvious in Figure 10 that the *z_b_* axis gyro error actually converges slowly at the beginning until the azimuth error has achieved a certain estimation accuracy in the practical application. From Figure 11, it is seen again that the *z_b_* axis gyro error converges much faster with the constrained KF scheme than by the conventional KF scheme. However, combining Figures 10 and 11, one can see that estimation of the azimuth error by the constrained KF scheme does not show better performance than by the conventional KF scheme at the beginning in practical applications. Compared to the estimation accuracy achieved by the conventional KF scheme, the better estimation of the azimuth error at the end of the filtering process by the constrained KF scheme has benefited from the better estimation of the *z_b_* axis gyro error. Looking through Figures 8, 9, 10, and 11, it can be seen that, to a certain extent, influences of ***P***(0) on the azimuth error and the *z_b_* axis gyro error will converge to zero with filtering carrying on.

### 4.2. Experimental Results

Then, multiple practical experimental tests can be done on this postulate system at a fixed position to compare the stationary north-finding performance possible with the above two filtering schemes.

**Figure 12.**
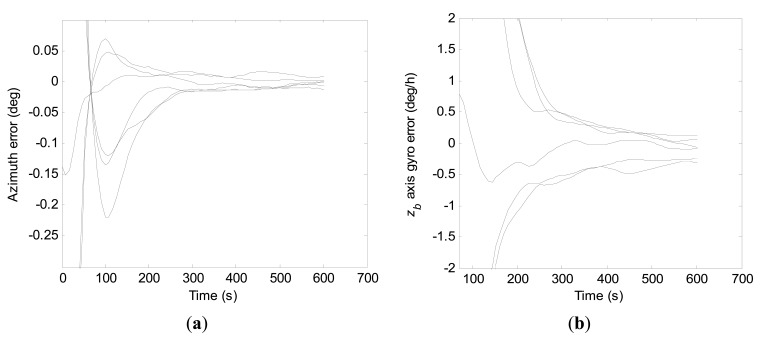
Convergence curves of the azimuth error and the *z_b_* axis gyro error within 600 s by the conventional KF scheme. (**a**) Azimuth error; (**b**) *z_b_* axis gyro erro.

Convergence curves of the azimuth error and the *z_b_* axis gyro error within 600 s north-finding time by the conventional KF scheme are shown in Figure 12, respectively. For purpose of clarity, vertical scale units of the figures are adjusted. Due to the severe fluctuation at the beginning, diagrammatic curves of the *z_b_* axis gyro error are given from 100 s. From the statistical evaluation, the 1-σ values of final azimuth error and final *z_b_* axis gyro error at 600 s are 22.8 arc-seconds and 0.18 deg/h, respectively.

**Figure 13.**
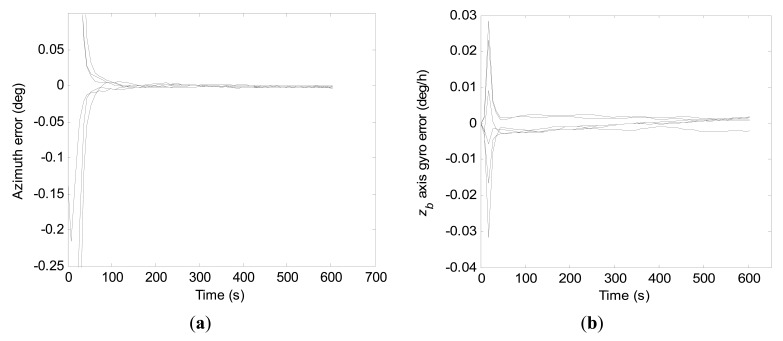
Convergence curves of the azimuth error and the *z_b_* axis gyro error within 600 s by the constrained KF scheme. (**a**) Azimuth error; (**b**) *z_b_* axis gyro erro.

Convergence curves of the azimuth error and the *z_b_* axis gyro error within 600 s north-finding time obtained with the constrained KF scheme are shown in Figure 13, respectively.

Apparently, as the added measurement quantity has improved the degree of observability of the *z_b_* axis gyro bias, the azimuth error and the *z_b_* axis gyro error have converged to steady value much faster by the constrained KF scheme than by the conventional KF scheme. Additionally, the 1-σ values of final azimuth error and final *z_b_* axis gyro error at 600 s are 4.4 arc-seconds and 0.0014 deg/h from the statistical calculation, respectively.

From Figures 12 and 13, it can be seen that the constrained KF scheme behaves better in north-finding performance than the conventional KF scheme. However, compared to results of the stochastic observability analysis with the *a priori* knowledge of the initial states, there is still potential to improve the estimation accuracy of *z_b_* axis gyro bias.

## 5. Conclusions

To improve the north-finding performance of the ARIMU on a stationary base, a constrained KF using a state equality constraint has been studied in depth. By analyzing the characteristics of the ARIMU on a stationary base, we obtained a linear state equality constraint to modify the conventional observation model. Taking advantage of its basic properties of intuitive linear-algebraic characterizations of the stochastic observability, analytical representations of estimation behaviors of the concerned navigation errors when implementing the conventional KF scheme and the constrained KF scheme during stationary north-finding were derived. Evidently, explicit formulations of navigation errors with influencing variables have helped us to maintain good physical insights into the estimation behaviors of the concerned navigation errors during stationary north-finding when implementing the schemes, which is one of the most significant advantages of the stochastic observability approach.

Multiple practical experimental tests at a fixed position were done on a postulate system to compare the stationary north-finding performance by the two filtering schemes. The experimental results show that the azimuth error and the *z_b_* axis gyro error have converged to a steady value much faster by the constrained KF scheme than by the conventional KF scheme. From our theoretical investigation and practical experimental verification of the ARIMU with ring laser gyros, the constrained KF scheme has on the whole demonstrated its superiority over the conventional KF scheme for the purposes of ARIMU stationary north-finding.
